# Smoking prevalence and attributable deaths in Thailand: predicting outcomes of different tobacco control interventions

**DOI:** 10.1186/s12889-019-7332-x

**Published:** 2019-07-23

**Authors:** Suchunya Aungkulanon, Siriwan Pitayarangsarit, Kanitta Bundhamcharoen, Chutima Akaleephan, Virasakdi Chongsuvivatwong, Ratsida Phoncharoen, Viroj Tangcharoensathien

**Affiliations:** 1 International Health Policy Program, Ministry of Public Health, Nonthaburi, Thailand; 2 Tobacco Control Research and Knowledge Management Center, Mahidol University, Bangkok, Thailand; 3 Epidemiology Unit, Faculty of Medicine, Prince of Songkla University, Songkhla, Thailand

**Keywords:** Tobacco, Policy, NCD global target, Thailand

## Abstract

**Background:**

Despite substantial positive impacts of Thailand’s tobacco control policies on reducing the prevalence of smoking, current trends suggest that further reductions are needed to ensure that WHO’s 2025 voluntary global target of a 30% relative reduction in tobacco use is met. In order to confirm this hypothesis, we aim to estimate the effect of tobacco control policies in Thailand on the prevalence of smoking and attributed deaths and assess the possibilities of achieving WHO’s 2025 global target. This paper addresses this knowledge gap which will contribute to policy control measures on tobacco control. Results of this study can help guide policy makers in implementing further interventions to reduce the prevalence of smoking in Thailand.

**Method:**

A Markov chain model was developed to examine the effect of tobacco control policies, such as accessibility restrictions for youths, increased tobacco taxes and promotion of smoking cessation programs, from 2015 to 2025. Outcomes included smoking prevalence and the number of smoking-attributable deaths. Due to the very low prevalence of female smokers in 2014, this study applied the model to estimate the smoking prevalence and attributable mortality among males only.

**Results:**

Given that the baseline prevalence of smoking in 2010 was 41.7% in males, the target of a 30% relative reduction requires that the prevalence be reduced to 29.2% by 2025. Under a baseline scenario where smoking initiation and cessation rates among males are attained by 2015, smoking prevalence rates will reduce to 37.8% in 2025. The combined tobacco control policies would further reduce the prevalence to 33.7% in 2025 and 89,600 deaths would be averted.

**Conclusion:**

Current tobacco control policies will substantially reduce the smoking prevalence and smoking-attributable deaths. The combined interventions can reduce the smoking prevalence by 19% relative to the 2010 level. These projected reductions are insufficient to achieve the committed target of a 30% relative reduction in smoking by 2025. Increased efforts to control tobacco use will be essential for reducing the burden of non-communicable diseases in Thailand.

## Background

Non-communicable diseases (NCDs) are the leading causes of death globally. In 2011, the United Nations General Assembly adopted a political declaration which committed its member states to focus on the prevention and control of NCDs [1]. Their goal is to achieve a 25% reduction of premature mortality due to NCDs by 2025 (the 25 × 25 target). The World Health Organization identified six targets for the prevention and control of risk factors for NCDs and two targets on availability and use of essential medicines and technologies. One of these targets is a 30% relative reduction in the prevalence of current tobacco use in persons aged 15 or more years between 2010 and 2025. Global modelling shows that tobacco use will have the largest effect in terms of reducing premature mortality from NCDs [2]. To plan and prioritize national tobacco control strategies, it is important for the Thai government to know whether or not the 30% target can be achieved and if not, what additional or enhanced policy interventions are needed to achieve the target [3]. This paper addresses this knowledge gap which will contribute to policy control measures on tobacco control.

NCDs have become a critical public health issue for Thailand. Deaths from NCDs accounted for 74% of the total 539,000 deaths in Thailand in 2016 and are predicted to continue to increase rapidly [4]. Tobacco use is an important modifiable risk factor as available interventions have been proven to be effective and 1 in 6 deaths from NCDs are caused by tobacco [5]. Smoking is the first leading risk factor for early death and disability in Thailand, particularly for people with cancer, pulmonary complications and heart disease. Almost 50,000 people die due to tobacco use each year [6]. The social costs of smoking were estimated at 2.18 billion US dollars, approximately 0.78% of the GDP, while the tobacco industry produced only 0.50% of the total GDP [7].

Having ratified the Framework Convention on Tobacco Control, Thailand has actively implemented many of the tobacco control policies which has shown remarkable success in reducing overall smoking rates from 23% in 2003 to 19% in 2017 [8, 9]. Despite some successes, challenges remain; for example, the increasing smoking prevalence rates among youth, the high proportion of roll-your-own cigarettes in adults, an ineffective regulation of tobacco sales to underage youths, second hand smoking in households, increasing gaps in the smoking rates between males and females, emergence of electronic cigarette use among youth, and difficulties in eliminating the illicit tobacco trade [10–13]. This suggests that the previous tobacco control policies might be less effective in some populations and/or not last very long. The Tobacco Products Control Act 2017 (BE 2560) entered into force on 4 July 2017. The law increased the minimum age for purchasing tobacco products from 18 to 20 years and updated the legislation on tobacco advertising, promotion and sponsorship among other regulations [14] This is expected to strengthen the prevention of youth smoking and secondhand smoking.

Despite a prior study in Thailand estimating that a 25% reduction in the prevalence of smoking during 1991–2006 was due to four main policies; increased cigarette taxes, smoke-free public areas, bans on advertising tobacco products, and the addition of warning labels on cigarette packs [15], we are not confident that the implementation of the Tobacco Products Control Act 2017 can encourage Thai residents to quit smoking, or deter them from initiating smoking, to such a level that would ensure the whole country achieves a 30% reduction in smoking prevalence by 2025.

In order to forecast the achievable level, this study uses a modeling method to project the smoking prevalence and attributable deaths in the previous policy context and six new scenarios including effects of the new law on youth smoking prevention, other feasible policies, and other effects of the Tobacco Product Control Act.

## Method

### Smoking model

We used a Markov chain model to estimate the projected smoking prevalence and attributed mortality in 2025. A discrete-time Markov chain is a stochastic process, which consists of a finite number of states and transition probabilities among the different states. In this study, the model begins by using data from the base year of 2015 with the population classified into three states: never, current, and former smokers stratified by 5-year age groups and gender.

**Fig. 1:**
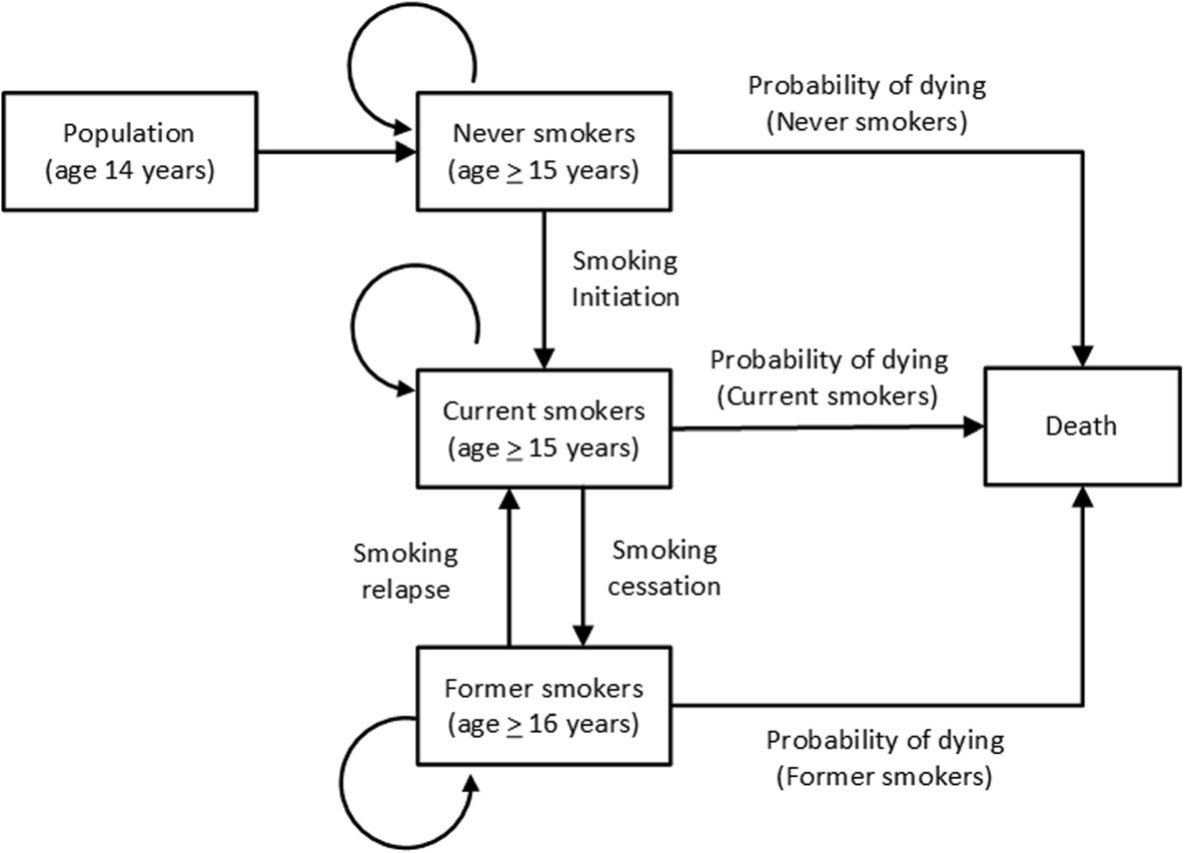
Overview of 2015 base Markov chain model used to predict the prevalence of smoking and attributed mortality in 2025

In the Markov model, death is the final absorbing state, a state in which people can enter but cannot leave. The prevalence of never, current and former smokers stratified by age and gender from 2015 to 2025 was estimated using four parameters: (1) the cessation rate among current smokers; (2) the smoking initiation rate; (3) the relapse smoking rate among quitters; and (4) the probabilities of death for each group. The populations aged 15 years or more, based on the Thai census, were entered into the model annually and assigned as never smokers. Figure 1 shows a transition diagram for each of the states in the model.

We developed two models which estimate the projected smoking prevalence and attributed mortality in 2025. The 2015 baseline model refers to the current smoking initiation and cessation rate. Several forecasting models were constructed to predict the effect of different interventions on the prevalence of smoking and attributed mortality. Table 1 presents the probabilities of moving from one state into another state for the next year.

**Table 1:** Transition matrix showing probabilities of movement from one state to another in the cycle of smoking behavior

Current year	Next year			
	Never smoker	Current smoker	Former smoker	Death
Never-smoker (NS)	1-initiation-pdying^a^ (NS)	Initiation	0	pdying^a^ (NS)
Current-smoker (CS)	0	1-cessation-pdying^a^ (CS)	cessation	pdying^a^ (CS)
Former-smoker (XS)	0	relapse	1-relapse-pdying^a^ (XS)	pdying^a^ (XS)
Death	0	0	0	1

^a^*pdying* probability of dying

### Data sources

The smoking prevalence, cessation rate, initiation rate, and other related parameters were drawn from the most recent smoking and drinking behavior survey conducted regularly by the National Statistics Office (NSO) and expressed as percentages. All-cause mortality data for 2015 was obtained from the Strategy and Planning Division, Ministry of Public Health. The mid-year population data before 2015 were obtained from Ministry of Interior and the population projections for years after 2015 were obtained from Office of the National Economic and Social Development Board. SAS software version 9.4 was used to analyze the data.

### Parameters

#### Smoking status

Current smokers were those who reported smoking either regularly or occasionally. Former smokers were those who reported ever smoking in their lifetime but were not current smokers.

#### Cessation rate

Annual cessation rates were measured as the number of current smokers in each year who became non-smokers the following year divided by the number of current smokers in each previous year. Cessation rates were estimated for each age and gender group.

$$ cessation\ rate=\frac{\mathrm{former}\ \mathrm{smokers}\ \mathrm{who}\ \mathrm{had}\ \mathrm{not}\ \mathrm{smoked}\ \mathrm{for}\ 12-23\ \mathrm{months}}{\mathrm{former}\ \mathrm{smokers}\ \mathrm{who}\ \mathrm{had}\ \mathrm{not}\ \mathrm{smoked}\ \mathrm{for}\ 12-23\ \mathrm{months}+c\mathrm{urrent}\ \mathrm{smokers}\ \mathrm{who}\ \mathrm{have}\ \mathrm{smoked}\ge 24\ \mathrm{months}} $$

#### Initiation rate

Annual initiation rates were measured as the number of current smokers in each year who did not smoke in the previous year divided by the number of non-smokers in the previous year. Initiation was modeled from age 15 to 24 years.

$$ initiation\ rate=\frac{\mathrm{current}\ \mathrm{smokers}\ \mathrm{who}\ \mathrm{have}\ \mathrm{smoked}<12\ \mathrm{months}}{\mathrm{current}\ \mathrm{smokers}\ \mathrm{who}\ \mathrm{have}\ \mathrm{smoked}<12\ \mathrm{months}+ never\ smokers} $$

#### Relapse rate

There are empirical challenges in measuring relapse rates. Since the survey did not contain a question asking for the specific year that a current smoker quit smoking, we used the annual incidence of smoking relapse after 1 year from a meta-analysis [16].

#### Probability of dying from smoking

The annual probabilities of dying among the never, current and former smokers were estimated from the age and gender-specific mortality rates. These mortality rates were calculated from 1) all-cause mortality rates from Thai population life tables for 2015, and 2) the relative risk of mortality from smoking from the American Cancer Society (ACS) Cancer Prevention Study phase two data (CPS-II) [17]. The formulas applied to estimate the probabilities of dying for never, current and former smokers are shown in the Appendix.

#### Model outcomes

The two primary outcomes from the model were annual smoking prevalence and smoking attributable deaths stratified by age and gender. We compared the effects of different tobacco control interventions with the 2015 baseline scenario. The cumulative number of lives saved was defined as the difference between the 10-year cumulative number of smoking attributable deaths from the 2015 baseline scenario and that calculated from policy interventions.

#### Tobacco control policy effects

The effects of control policies were assessed based on the effectiveness of tobacco control interventions, which was based on the number of smokers who quit, the number of non-smokers prevented from initiating smoking, and the coverage of the target population under the control policy. For example, the effect of cessation policies on increased cessation rate is equal to 1 + (intervention coverage rate × success rate).

### Scenarios of tobacco control interventions

#### Baseline scenario

In the 2015 baseline scenario, age-specific initiation and cessation rates were estimated from the smoking and drinking behavior survey conducted by the NSO. We assumed that initiation and cessation rates remained constant throughout the projection period between 2015 and 2025.

#### Scenario 1: policies involving restricting access to tobacco among youth

Prohibition of tobacco sales to youths under the age of 18 years was implemented in 1992. In 2017 the minimum legal age for purchasing cigarettes was increased to 20 years. However, monitoring, surveillance and enforcement are largely ineffective as recent studies have reported that more than half of smokers aged under 18 years buy cigarettes [18]. Effective interventions in prohibiting smoking include increased taxes and retail prices of tobacco products beyond the youths purchasing capacity, anti-smoking mass media campaigns, smoke-free policies, school curricula on the mortality impact of tobacco, and restricting minors from purchasing tobacco-related products [19, 20]. These policies have already had a substantial impact in Thailand. Further gains might be realized by implementing stricter youth access policies. Our model assumed that youth access interventions could reduce the smoking initiation rate among adolescents by a maximum of 50%, and this effect remained constant over the entire projection period.

#### Scenario 2: annual increases of tobacco taxes and prices by 15%

Most studies in low- and middle-income countries have shown that a 10% increase in the price of cigarettes will reduce consumption by 4–6% with younger smokers being more price-sensitive than older smokers [21]. Empirical evidence suggests that about half of the reduction in cigarette consumption is the result of current smokers quitting altogether and another half results from the remaining smokers reducing their smoking frequency [22]. However, price effects on smoking initiation among youths have been found to have little impact [23].

Regular price increases above the inflation rate and purchasing power are effective in curbing smoking demand [24]. One study in 2009 found that despite an increase of cigarette prices by 8.7% in real terms, cigarette consumption actually increased [25]. In this study, in line with experts’ recommendations, we assumed a 15% annual increase in tobacco tax and applied a − 0.5 price elasticity of smoking consumption. We further assumed a double effect on young adults aged 15–24 years. Therefore, the model specification is that a 15% annual price increase will increase the cessation rate by 3.75% among those aged 25 years or more and 7.5% among those aged less than 25 years.

#### Scenario 3: increased coverage of a national telephone quit-line

Establishment of the Thailand National Quit-line center in 2009 expanded smokers’ access to behavioral counseling services to help them quit smoking. A well-publicized, multi-session proactive quit-line was estimated to reach between 1 and 4% of smokers over a 1 year period [26–28]. Coverage of the quit-line is estimated to reach 6–8% of all smokers [29]. The International Tobacco Control (ITC) Survey reported that globally, Dutch smokers had the highest use of a quit-line (around 12% of smokers) while in Thailand the figure was only 3% [30]. Cochrane Systematic Reviews estimated that a quit-line can increase the smoking cessation rates by between 20 and 36% [31]. In this study, we used data from a study by Meeyai which reported a 12-month effectiveness of 19.5% for Thailand’s smoking cessation quit-line [32] and assumed effects remained constant over the projection period. It was further assumed that the intervention can reach a maximum of 12% of current smokers.

#### Scenario 4: increased coverage of a health facility-based brief advice intervention on smoking cessation

Brief advice is one of the most cost-effective disease prevention interventions [33], and is feasible to integrate into primary health care centers due to its relatively low cost. Cochrane Systematic Reviews estimated that a brief advice intervention can increase the rate of smoking cessation by up to 66% [34]. Despite these guidelines and its cost-effectiveness, this intervention is largely neglected by health professionals in low- to middle-income countries. Smokers are not offered cessation advice in clinical encounters [35]. The ITC Survey reported large variations in coverage of brief advice by health professionals, ranging from less than 10% in New Zealand to over 50% in the USA [30]. Thailand’s Health Welfare Survey reported that 29% of the population had visited a health facility during the past 30 days prior to the survey. We assumed that 30% of smokers visit a healthcare provider of which all received advice to quit smoking, and the smoking cessation rate from this intervention was 66%.

#### Scenario 5: combined effects of policy interventions

When more than one policy is in effect, the rates of reduction in smoking initiation and cessation can be multiplicative, implying that the effect of an additional policy is synergized and increases the effect of another policy.

#### Scenario 6: theoretical scenarios

In the theoretical scenario, we assumed that the smoking initiation intervention could reduce smoking initiation in youths and young adults to zero. We also assumed that 100% of the current smokers were covered by the cessation interventions using the telephone quit-line and health facility based brief advice offered by health professionals.

### Model validation

To test the validity of our estimates, smoking rates predicted by the model were compared to the observed prevalence rates from national smoking and drinking survey data reported by the NSO. We use these models to project the gender-specific smoking prevalence for the period between 2007 and 2014 compared with the survey data in 2007 and 2011 on smoking prevalence.

## Results

### Validation: predictions of smoking prevalence

The predicted prevalence of smoking for males in 2007, 2011, and 2014 were similar to the prevalence from the smoking and drinking behavior survey and showed a similar pattern of decline. For females the smoking prevalence was 2.4% in 2007, 2.1% in 2011, and 2.2% in 2014. Due to the low prevalence of female smokers, this study applied the model to estimate the smoking prevalence and attributable mortality among males only.

### Baseline scenario: input parameters and projection of smoking prevalence

#### Smoking prevalence

In the smoking and alcohol consumption behavior survey, the prevalence of current smoking for those aged 15 years and over was 40.8% (95% confidence interval [CI]: 39.4–41.8) among males and 2.2% (95% CI: 1.9–2.5) among females. Table 2 shows estimates of the national prevalence of never, current, and former smokers stratified by age and sex.

**Table 2:** Prevalence of smoking profiles (%) by gender and age group, Thailand, 2014 household survey

	Males			Females		
	Never smokers	Current smokers	Former smokers	Never smokers	Current smokers	Former smokers
Overall	46.7	40.5	12.8	96.7	2.1	1.2
Age group						
15–19	79.1	19.4	1.4	99.2	0.3	0.5
20–24	54.2	41.5	4.3	98.6	0.2	1.2
25–29	41.9	51.3	6.8	98.7	0.9	0.4
30–34	47.4	46.4	6.3	98.4	1.3	0.4
35–39	44.7	46.9	8.4	97.3	1.8	0.9
40–44	43.7	45.0	11.3	96.7	2.3	1.0
45–49	40.1	48.4	11.5	96.8	2.9	0.3
50–54	41.4	41.2	17.4	96.1	3.1	0.8
55–59	41.0	41.8	17.2	94.3	4.2	1.5
60–64	35.4	38.7	25.9	94.7	3.3	2.0
65–69	36.4	33.8	29.8	94.0	3.3	2.8
70–74	38.9	32.3	28.7	96.0	1.4	2.7
75+	41.1	23.4	35.6	91.9	4.1	4.0

Source: Estimates from the smoking and drinking behavior survey, National Statistics Office

**Fig. 2:**
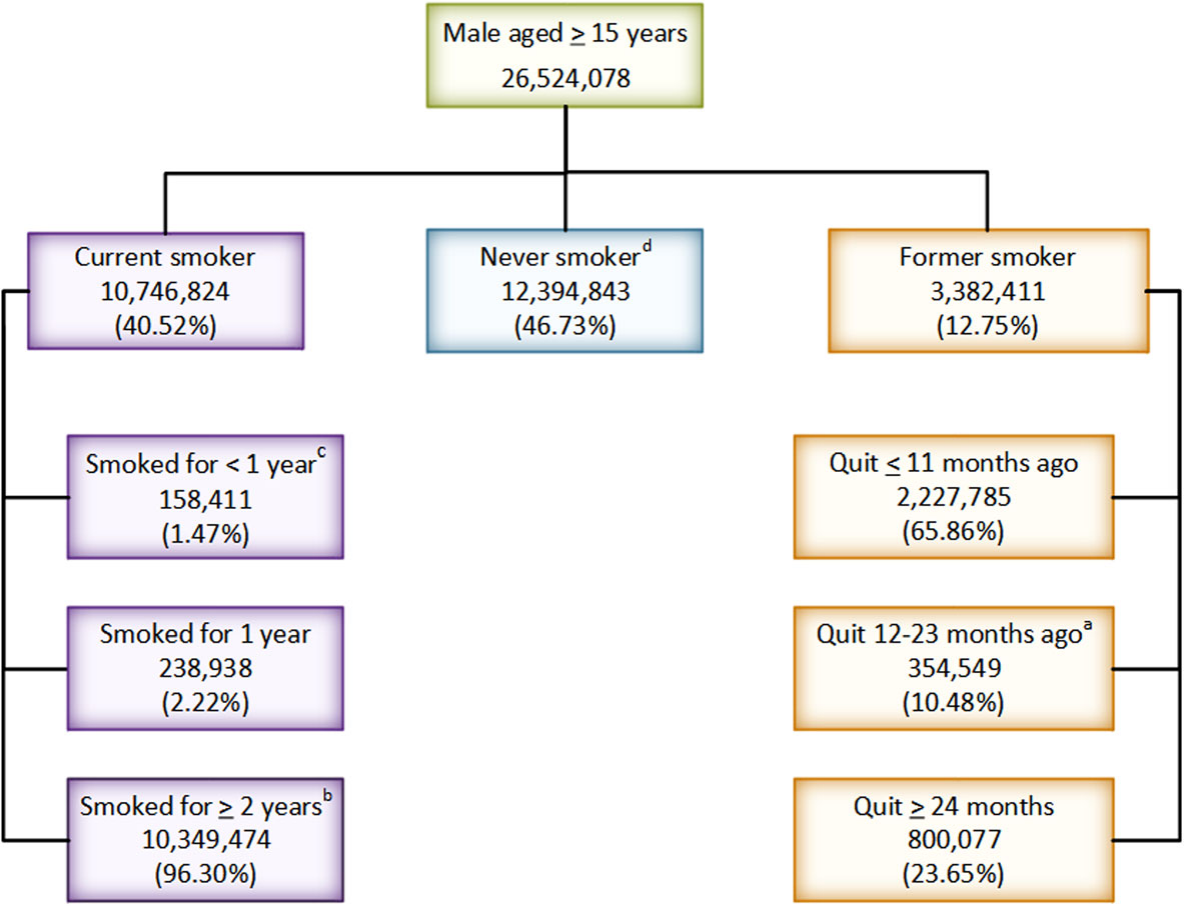
Number of smokers and quitters among males aged 15 years and over stratified by smoking duration, 2014 (Annual cessation rate = a/(a + b); Annual initiation rate = c/(c + d))

Figure 2 shows the number of smokers and quitters in 2014 among males aged 15 years and over stratified by smoking duration for which the annual cessation and initiation rates are estimated.

#### Annual cessation rate

In the tobacco consumption survey, there were 3.4 million former smokers of which 65.9, 10.5 and 23.7% had quit smoking for less than 12 months, between 12 and 23 months, and 24 months or more, respectively. The annual smoking cessation rate among male smokers was 3.3%. As shown in Table 3, rates increased with increasing age with the lowest cessation rate of 1.5% found in those aged 20–34 years.

**Table 3:** Annual cessation rates in males stratified by age group, 2014

Age group	Annual cessation rate
15–19	3.3%
20–24	1.4%
25–29	1.5%
30–34	1.3%
35–39	2.2%
40–44	2.2%
45–49	3.0%
50–54	3.8%
55–59	6.1%
60–64	4.8%
65–69	6.1%
70–74	7.4%
75+	13.6%
All ages	3.3%

#### Annual initiation rates

The annual smoking initiation rates among males aged 15–19, 20–24, 25–29 and 30 or more years were 5.0, 2.7, 0.8 and 0.1%, respectively.

#### Prediction of smoking prevalence under baseline scenario

**Fig. 3:**
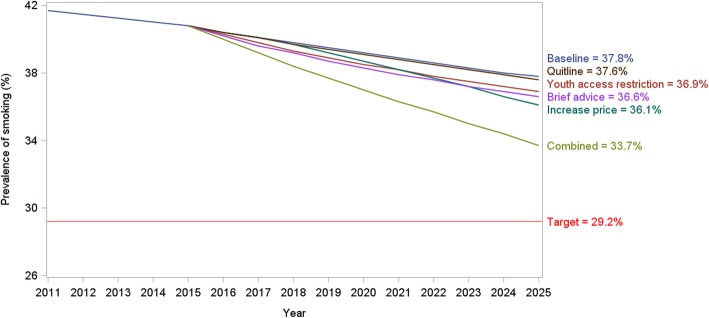
Effects of different smoking control policies on smoking prevalence in male, 2015 to 2025

Figure 3 shows the trend in smoking prevalence for males. Under the baseline scenario in which cessation and smoking initiation rates continued unchanged, the smoking prevalence in males is expected to decrease from 40.8% in 2015 to 37.8% in 2025. Despite this reduced prevalence, the estimated number of current smokers will remain over 10 million due to population growth.

### Effects of tobacco control policies on smoking prevalence and deaths

The projected annual smoking prevalence under each of the six scenarios is shown in Fig. 3. From the baseline scenario, the annual 15% increase in cigarette tax would have the highest contribution (compared with other single interventions) to reducing the male smoking prevalence, with a reduction from 41.7% in 2010 to 36.1% in 2025, which is equivalent to a 4.5% reduction against the 2025 baseline prevalence or a 13.4% reduction compared to 2010. The synergistic effects of the combined interventions would reduce the prevalence to 33.7% in 2025, which is equivalent to a 10.8% reduction against the 2025 baseline or a 19% relative reduction from 2010.

While an annual tax increase had the largest impact on smoking prevalence, brief advice showed potent and immediate effects on reduction in smoking attributable deaths with almost 55,000 fewer deaths. Combined interventions resulted in a 4% reduction. As shown in Table 4, the cumulative number of deaths over the 10 year period between 2016 and 2025 was 2,235,851 among males aged > 15 years. By 2025, a total of 99,784 tobacco related deaths, or 4.5% of the total deaths from all causes, were projected to be averted by implementing the combined tobacco policies. This follows the brief advice to quit smoking in clinical settings which contributes to 2.46% of all-cause mortality.

**Table 4:** Number of baseline and smoking attributed deaths averted from different tobacco control policies, 2016–2025

Year	Baseline all-cause deaths (aged > 15 years)	Restricted access to youth	Price increase	Quit-line	Brief advice	Combined
2016	256,947	0	0	0	0	0
2017	248,268	0	0	0	0	0
2018	240,143	78	0	78	640	811
2019	232,517	223	116	206	1,686	2,290
2020	225,346	425	423	366	2,982	4,332
2021	218,591	674	967	545	4,426	6,856
2022	212,219	963	1,776	734	5,950	9,804
2023	206,200	1,286	2,866	930	7,507	13,127
2024	200,505	1,634	4,244	1,126	9,069	16,790
2025	195,115	2,001	5,909	1,320	10,612	20,759
Cumulative	2,235,851	9,668	24,164	6,816	54,999	99,784
Reduction in mortality (%)		0.43%	1.08%	0.30%	2.46%	4.46%

**Fig. 4:**
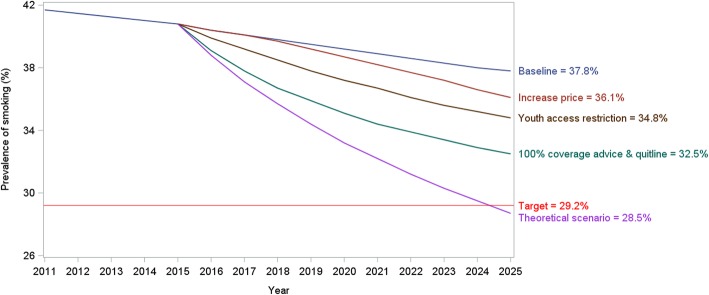
Effect of smoking control policies on smoking prevalence in males aged 15 years or more under a theoretical ideal scenario, 2015 to 2025

Figure 4 shows the effects of each theoretical scenario on the prevalence of smoking. The projected smoking prevalence under an ideal scenario (maximum combined interventions) in 2025 would reduce the prevalence to 28.5%.

## Discussion

This study applied a Markov chain model to predict the smoking prevalence and attributed deaths in 2025 among males aged 15 years or more based on the impact of different tobacco control policies. Under a baseline scenario where no additional intervention is implemented, the smoking prevalence in males would reduce from 40.8% in 2015 to 37.8% in 2025.

The most effective single policy for reducing the male smoking prevalence was found to be a 15% annual increase in tobacco tax, a policy which would reduce the prevalence by 4.5% in 2025. Implementation of a comprehensive combination of all tobacco control policies would reduce the male smoking prevalence by 10.8% in 2025 and approximately 100,000 deaths could be averted, which is equivalent to a 4.5% reduction in all-cause mortality. The comprehensive combination of tobacco control policies could reduce the male smoking prevalence by 19% in 2025, which is well below the global target of 30%.

From 2015 to 2025, the male smoking prevalence in Thailand would decrease from 40.8 to 37.8% (0.3 percentage points per year) given no additional intervention. Our finding confirms a study by WHO where the male smoking prevalence was projected to decrease from 42.7% in 2015 to 39.5% in 2025, equivalent to an annual reduction of 0.32 percentage points [36]. We projected that combined tobacco control policies would lead to as much as a 19% relative reduction in the male smoking prevalence by 2025. The only way to achieve this 30% reduction would be to assume a 100% smoking cessation rate and a zero smoking initiation rate, both of which are implausible.

Taxation on tobacco products has been an effective means of tobacco control in many countries. Our findings are consistent with a recent systematic review of over 100 studies, including a growing number from low- and middle-income countries [21]. This study shows that a 15% annual increase in cigarette tax yields the highest reduction in smoking prevalence, although such a high tax increase would require extraordinary political leadership amidst a tobacco industry that exerts staunch resistance and political lobbying [37]. The consequences of raising cigarette taxes, such as increased cigarette smuggling and consumption of lower priced cigarettes, [25, 38] would likely dilute the intended effects of this intervention. The tobacco industry takes advantage of this loophole in the ad valorem excise tax system in Thailand to falsely declare a lower imported price. This dilutes the impact of an increased retail price [39]. To overcome this loophole, the actual retail prices should be used as the basis for the ad valorem taxation. This means the tax rate should be based on the retail price, not the reported ex-factory or imported price. A combination of ad valorem and specific tax per weight or per cigarette would have a stronger impact on price increases. The Thai government should review the tax rate regularly to keep pace with inflation and the increased capacity of smokers to pay for cigarettes [40].

This study demonstrates that professional brief advice is the second-best policy for reducing the male smoking prevalence and also contributed most to the reduction of smoking attributed deaths. Evidence suggests that a few minutes of brief advice given by health professionals at any clinical encounter can increase the smoking cessation rate [34]. A professional-led brief advice session informing smokers about the harms of smoking is technically very feasible under the universal health coverage system in Thailand as there is a high level of clinical contact between patients and health professionals (Thailand has more than 3.5 outpatient visits per capita per year and an admission rate of more than 11%) [41]. There are also more than a million village health volunteers throughout the country. The cost of implementing these advice sessions should be low as they can easily be integrated into existing counseling sessions by health professionals in clinical settings and are well supported by the village health volunteers.

The quit-line program provide behavioral counselling to help callers (smokers and their relatives) to develop and follow a plan to quit smoking. However, it was found have the least impact on reducing the all-cause mortality due to smoking and the program has yet to scale up its performance and coverage of services. New information technologies and social media allow for online transmission of short messages related to quitting and an info-graphic quit smoking program that might increase coverage to half of all target smokers. This policy option should be explored further and implemented at the appropriate scale, not just for Thailand, but for other low- to middle-income countries where there is an inadequate public health infrastructure.

Prevention of smoking initiation among youth is a key to ending the tobacco epidemic in Thailand. We have projected that preventing smoking onset in youth and young adults would reduce the male smoking prevalence in 2025 by 8%. However, the unforeseen consequence of a policy to restrict under-age youth accessing tobacco products has shifted to other means of access such as receiving tobacco from relatives, friends, or even strangers [42]. The 2015 Global Youth Tobacco Survey reported that half of the underage youth had no difficulty in buying cigarettes, reflecting weak regulatory environments and enforcement capacities [13]. Although electronic cigarettes remain illegal in Thailand, 3.3% of youth were current e-cigarette smokers [13]. These results indicates that enforcement of the existing laws is weak, not only in Thailand, but around the world.

Our comparison of the theoretical ideal and the realistic scenario on smoking cessation interventions illustrated the importance of population coverage. When the quit-line and brief interventions fully cover all smokers, the prevalence reduced by 4 percentage points, although there was a 3.4 times increase in cessation rate (from 22 to 99.2%).

### Limitations of the study

Several of our methodological assumptions are likely to have had an effect on the study outcomes. The policy effect sizes depend on underlying assumptions, such as initiation and cessation rates, which both affect the smoking prevalence. Predictors of successful quitting include a lower level of nicotine dependence, a longer duration of time since the last attempt to quit smoking, a higher level of self-efficacy, and an absence of pressure from peer smokers.

Our estimates did not include initiation of new tobacco products, such as electronic cigarettes and policies on smoke free interventions, such as mass media campaigns, advertising bans, and package warning labels. Although these interventions appear to have raised awareness about the dangers of tobacco use, it is difficult to quantify the degree of these effects on tobacco use behavior and health outcomes.

Our results were possibly underestimated because females, due to their low smoking prevalence, were excluded from the analysis. However, the male smoking prevalence is about 20 times that of females. Because of their far greater consumption of tobacco, male smokers have disproportionately higher morbidity and mortality rates.

The excess risk of dying from smoking is a simple model based on the relative risk for current and former smokers. In reality, smoking-related mortality depends on many factors. Our method has low precision in predicting the smoking prevalence because of the limitation in estimating initiation and cessation rates. The relative risks for mortality from smoking were based on data from a US Cancer Prevention study. American mortality risks and tobacco epidemiology may differ from that of the Thai population.

The impacts of tobacco control interventions differ according to demographic and socioeconomic characteristics [43], but these effects are generally reported as national averages across the entire population. Existing studies do not distinguish variation of impacts across population sub-groups. To address this issue, future studies should include the assessments of the impacts on different socioeconomic and geographic parameters.

## Conclusion

Despite modeling limitations, this study confirms that a 15% cigarette tax increase has the most impact on reducing the male smoking prevalence while brief advice could avert the highest number of smoking related deaths by 2025. Combined interventions have a synergistic effect with an estimated 99,800 deaths being averted between 2015 and 2025. Despite the decreasing prevalence, the reductions are projected to be insufficient to achieve a 30% reduction in the prevalence of tobacco smoking by 2025. Increased efforts to control tobacco use will be essential for reducing the burden of non-communicable diseases in Thailand.

## Data Availability

The data that support the findings of this study are available from National Statistical Office but restrictions apply to the availability of these data, which were used under license for the current study, and so are not publicly available. Data are however available from the authors upon reasonable request and with permission of National Statistical Office.

## Appendix

Relative risks (95% confidence intervals) of all-cause mortality by smoking status, data from Cancer Prevention Study phase II

**Table:**

	Males	Females
Never smoker	1	1
Current smoker	2.33 (2.26–2.40)	2.08 (2.02–2.14)
Former smoker	1.42 (1.38–1.45)	1.33 (1.29–1.37)

Source: American Cancer Society, Cancer Prevention Study phase two [8]

The following formulas were applied to estimate the probabilities of dying for never, current and former smokers.

$$ {M}_x^n=\frac{M_{x,t}^p}{RR_x^c\times {Prev}_x^c+{RR}_x^f\times {Prev}_x^f+1\times 1-\left({Prev}_x^c+{Prev}_x^f\right)\ } $$

Where

$$ {M}_{x,t}^c={RR}_x^c\times {M}_x^n $$

$$ {M}_x^f={RR}_x^f\times {M}_x^n $$

${M}_x^n$, ${M}_x^c$, ${M}_x^f$ = mortality rate for never (*n*), current (*c*) and former (*f*) smokers, respectively in age group *x*.

${M}_x^p$ = mortality rate of the total population (*p*) in age group *x*.

${Prev}_x^c$, ${Prev}_x^f$ = prevalence of current and former smokers, respectively in age group *x*

${RR}_x^c$, ${RR}_x^f$ = relative risk of mortality in current and former smokers, respectively compared to never smokers in age group *x*.

The mortality rates (MR) for never, current and former smokers for age group *x* were then converted into the probability of dying for age group *x* using the formula:

$$ {\mathrm{P}}_{\mathrm{x}}=1-{\mathrm{e}}^{-\mathrm{MRx}} $$

## Abbreviations

CI: Confidence interval
CS: Current smoker
NCDs: Non-communicable diseases
NS: Never smoker
NSO: National Statistics Office
XS: Former smoker
